# Doctoral Contribution to Nursing Science in Sub-Saharan Africa: A Document Review

**DOI:** 10.1177/08943184231207380

**Published:** 2023-12-06

**Authors:** Champion N. Nyoni, Yvonne Botma, Lizemari Hugo-van Dyk

**Affiliations:** 1 University of the Free State, Bloemfontein, Free State, South Africa

**Keywords:** doctoral education, nursing science, sub-Saharan Africa

## Abstract

Nursing science is at serious risk of extinction. The lack of funding for research, absence of healthcare policies underpinned by nursing science, and general lack of understanding of nursing metaparadigms all contribute to the stunted growth in nursing science. Doctoral research is a platform for the development and refinement of nursing science. The purpose of this qualitative retrospective document review was to describe the doctoral contribution to nursing in sub-Saharan Africa (SSA). Electronic dissertations for doctoral degrees in nursing within a 5-year period from universities in SSA were included. The extracted data comprising the purpose of the studies, the models used in the studies, and the studies’ contributions to nursing science were analyzed against a knowledge contribution framework. In total, 166 documents were included, mostly from South African universities, with a predominant focus on developing models, frameworks, and strategies within nursing practice. Only 17% of the studies applied grand nursing theories or models, with the rest of the studies applying theories from other disciplines. The contribution to nursing science from the doctoral studies was poor. The low uptake of nursing models in doctoral research in SSA may significantly contribute to the lack of refinement of nursing science within SSA. Structured approaches focused on integrating the nursing metaparadigms, theories, and models and fundamental underpins for doctoral education in Africa are essential to influencing the refinement of nursing science.

Nursing science is at serious risk of extinction (Barrett, 2017). The stunted growth in the refinement of the nursing paradigm, limited research in nursing science, and narrow Western-influenced worldviews of nursing contribute to the increased risk of extinction of the scientific discipline of nursing (Parse, 2015). Nursing science aims to enhance the understanding of human experiences through expanding nursing discipline–specific knowledge generated from empirical research and other methods of inquiry (Parse, 2015). Nursing metaparadigms, theories, and models align with nursing science and are nested within the four central phenomena of nursing: person, health, environment, and nursing (Donaldson & Crowley, 1978). The nursing metaparadigm, theories, and models demarcate the boundaries of nursing, its uniqueness from other disciplines, and contributions to society. However, Barrett (2017) explains that nursing science is often not clear to nurses, compromising the unique theoretical and practical contribution of nursing and nurses to society.

The limited growth and poor understanding of nursing science contributed to practical challenges in nurse education, practice, administration, and research (Pickler & Dorsey, 2022). Nurse education programs are predominantly inspired by medical models underpinned by behaviorist philosophies (Armstrong & Rispel, 2015; Robinson-Lane, 2021). Reese and colleagues (2016) explained that nursing practice continues to be executed through a series of unrelated tasks guided by algorithms prepared by other disciplines. The protracted shortage of the health workforce spliced with poorly understood nursing paradigms creates “task-shifting” opportunities where tasks from other health professions are shifted to nurses, often beyond their professional scope (Shumbusho et al., 2009). Healthcare environments favor lowly qualified nurses, who are hired to perform a limited set of skills and are then poorly remunerated, perpetuating a subservience normative (World Health Organization, 2020). Research in nursing science competes with other powerful fields, such as infectious diseases, cancer, and cardiology, which are often heavily funded (Reese et al., 2016), resulting in the fading of nursing models in academic discourse (Parse, 2016). Nurse scientists, who are expected to advance research in nursing science, ultimately are involved in developing and contributing to other disciplines.

Nurses compose the largest proportion of the health workforce within sub-Saharan Africa (SSA; World Health Organization, 2020). However, there are reported challenges in the adoption and assimilation of nursing models in mainstream healthcare, resulting in tensions related to the professional role of nurses (Agyeman-Yeboah et al., 2017). Nurses therefore find themselves engaged in basic healthcare tasks and appropriating medical responsibilities, which are often not aligned with the nursing science. Ultimately, nurses struggle to define and engage in what is genuinely nursing, thus muddying their practical contributions to healthcare. The fact that most of the grand nursing theories are Western influenced further complicates the professional definition of nursing, adding to the lack of clarity on the nursing role and limiting nurses’ scientific contribution from regions such as SSA. Robinson-Lane (2021) and Barrett (2017) argued for developing contextualized nursing science through testing, applying, and developing nursing-specific models, theories, and frameworks within specific contexts. The advent of doctoral education in nursing in SSA is an opportunity to develop and refine nursing science within SSA.

Doctoral research should culminate in a significant original contribution to knowledge for a given discipline (Australian Qualifications Framework Council, 2013). New ways of explaining or understanding phenomena, new knowledge, and even a new perspective on existing literature have all been described as entailing a “significant contribution.” The design science research (DSR) knowledge contribution framework posits four domains that classify knowledge contribution (Gregor & Hevner, 2013). These domains are based on the maturity of the research problem and that of the solutions, and they are interwoven to categorize knowledge contribution as (a) routine design, (b) improvement, (c) exaptation, and (d) invention (Table 1). Doctoral research in nursing can thus make a knowledge contribution to the field in the context of SSA.

**Table 1.** Domains in the DSR Knowledge Contribution Framework.

Domain	Description
Routine design	Apply known solutions to known problems (no significant knowledge contribution)
Improvement	Develop new solutions for known problems (research opportunity and knowledge contribution)
Exaptation	Extend known solutions from other fields to new problems (research opportunity and knowledge contribution)
Invention	Invent new solutions for new programs (research opportunity and knowledge contribution)

*Note.* DSR = design science research.

Doctoral qualifications in nursing are considered to be unique since they focus on nursing theory development, research, and practice in nursing science (Parse, 2015). However, the doctoral contributions to nursing science from the SSA context are unknown. In this article, we describe the doctoral contribution to nursing science in SSA through a document review. As we argue, doctoral research in nursing has a role to play; it must shape the development and refinement of nursing science. Insights into the doctoral contribution to nursing science in SSA could influence the discourse toward the development of contextualized nursing science.

## Material and Methods

### Design

A qualitative retrospective document review was conducted, as underpinned by the document analysis framework by Morgan (2022). This process involved specifying the review question and the population of interest, sampling and searching for documents, extracting data, and applying qualitative analysis to answer the review question—specifically,

What is the contribution to nursing science by doctoral research from SSA?

### Population and Sampling

This study included documents in the form of theses or dissertations submitted to universities based in SSA for the doctoral qualification in nursing. Inclusion criteria stipulated that these documents were submitted between January 2015 and December 2019 and that various names of the doctoral qualification in nursing were acknowledged. Specifically, we included dissertations submitted for the doctor of philosophy in nursing, doctor of philosophy in nursing science, philosophiae doctor in nursing, or doctor of nursing. We included all documents accessible online or through electronic repositories. We excluded documents beyond the timelines, in non-English language, and by nurses for nonnursing qualifications, including midwifery. Census sampling was applied to include all documents that met the inclusion criteria.

### Search Methods

Searching for the documents followed a stepwise process that started with identifying universities with nursing or nursing science departments, schools, faculties, or units. We searched for and created a list of all the English-speaking public universities in SSA with nursing or nursing science departments, schools, faculties, or units that offer at least a degree in nursing. Each university was investigated to determine if it provided doctoral qualifications in nursing programs through its university website. A finalized list of universities offering doctoral degree programs in nursing in SSA was shared with an information specialist, who supported the process by searching for documents within the 5-year timeline. All documents were accessed and retrieved through university libraries and additional online repositories. We screened the accessed documents against the inclusion criteria. A password-protected electronic platform was used to store all included documents, which we all accessed.

### Data Extraction

An author-generated tool supported the data extraction process, including the following elements: the region where the study was reported, the country where the study was conducted, the universities where the degrees were conferred, the focus of the study titles, the purpose of the study, the research designs and models used, and the described contribution of the doctoral research. We piloted the data extraction tool, and no amendments were made secondary to the pilot. With a research assistant, we extracted data from the documents into a Google form. The extracted data were then exported to a Microsoft Excel spreadsheet.

### Data Analysis

An inductive multistep process was applied in analyzing the data (Morgan, 2022). Frequencies were used to analyze the regions, countries, and universities where the studies were conducted. The four domains of nursing—namely, nurse education, nursing research, nursing practice, and health services administration (Reese et al., 2016)—were used to classify the titles of the studies, and frequencies were calculated per domain. As described in the documents, the purposes of the studies were inductively themed and frequencies tallied. Regarding the extracted theories, we initially classified them by their primary discipline of origin and subsequently calculated the frequencies of the disciplines. A similar approach was used to analyze the contribution to sciences as reported by the authors of the studies, where raw descriptions of contributions were clustered in categories based on their similarities. The categories were further mapped against the four domains of the DSR knowledge contribution framework (Table 1; Gregor & Hevner, 2013). A secondary analysis was conducted on the 26 documents that included nursing-specific grand, process, or middle-range nursing theories or models to underpin their doctoral studies. This analysis focused on identifying and classifying the nursing-specific theories for a comprehensive understanding related to nursing science.

### Ethics

The Health Science Research Ethics Committee of the University of the Free State approved this review (UFS-HSD2020/1069/2807). We purposely included documents that were available in the public domain. Personal identifiers such as names and student numbers were omitted to maintain confidentiality, although the doctoral graduates and their supervisors would be able to identify the titles of their work. Care was taken not to evaluate the dissertations’ quality but to focus on the document review question. We maintained high standards of ethics regarding accessing, analyzing, and storing the data.

### Rigor

The documents in this study were collected from multiple institutions within the SSA region through a transparent process that clarified inclusion and exclusion parameters a priori. We engaged established research models and approaches in the design, analysis, and reporting of this work. A pilot exercise was conducted to ascertain the validity and feasibility of the data extraction tool. In the pilot exercise, we sampled three dissertations, individually extracted the data from the same dissertation, and compared outcomes. Discussions assisted us in refining the data extraction tool. Given our experience in nursing research and secondary data analysis, we agreed on the extracted data, analysis approach, and reporting strategies.

## Results

In total, 166 documents were included in this document review. Most studies were conducted in the Southern African region (*n* = 142), followed by East Africa (*n* = 19) and West Africa (*n* = 4). Sixty percent of the studies were conducted in South Africa. In contrast, 15 studies were conducted in Ethiopia, 11 in Nigeria, and a few were conducted in other countries (see supplementary file 1). The University of South Africa (UNISA) and the University of Western Cape, both based in South Africa, were responsible for a combined 55% (*n* = 91) of documents in this review. Other universities had <15 documents included; notably, the University of Nairobi, in Kenya, was the only university with a document included outside South Africa. The following section presents the analysis outcomes based on the focus of dissertation titles, models that underpin the studies, and the contribution from the doctoral studies.

### Focus of the Studies

The studies appeared to focus on nursing practice (51%, *n* = 85), health services administration (28%, *n* = 47), and nurse education (20%, *n* = 33), with one study focused on nursing research. The titles of the studies were aligned with the purpose of their research. Most studies focused on developing artifacts in the form of frameworks, models, or guidelines (*n* = 97), as well as exploring phenomena (*n* = 42), evaluating interventions (*n* = 13), and determining factors influencing phenomena (*n* = 14). Qualitative research (*n* = 53) was the predominant research approach.

### Theories Used to Underpin Studies

Various theories underpinned the research in the included studies. These theories were classified by their disciplinary origins and analyzed through frequencies. Most studies applied education and learning-related theories (*n* = 27), followed by nursing-related theories (*n* = 26) and then administration and leadership theories (*n* = 24) . Theories related to healthcare systems and others classified as *miscellaneous* or *own* theories were in 44 documents. Health promotion theories (*n* = 18) and theories from the disciplines of sociology (*n* = 13) and psychology (*n* = 7) were included. Only seven studies did not report a theory underpinning their research (Table 2).

**Table 2.** Classification of Theories in Studies.

Classification	*n* (%)
Education and learning	27 (16)
Nursing	26 (17)
Administration and leadership	24 (14)
Healthcare systems	22 (13)
Miscellaneous/own	22 (12)
Health promotion	18 (11)
Sociology	13 (8)
No specified theory	7 (4)
Psychology	7 (4)

### Contribution From the Documents

Most studies described contributions as models, guidelines, or frameworks for either clinical practice (*n* = 44) or implementation of clinical (*n* = 16) and educational (*n* = 16) interventions. Other contributions focused on professional development (*n* = 26), practice environments and administration (*n* = 19), educational programs for students (*n* = 11), and educational programs for patients (*n* = 4). Two studies described their contribution as tools, while the contribution from 14 dissertations was classified under *other* (Table 3).

**Table 3.** Contribution to Science.

Contribution	*n* (%)
MGF clinical practice	44 (27)
MGF implementing clinical interventions	28 (17)
Professional development	26 (16)
Practice environments and administration	19 (11)
MGF implementing education interventions	16 (10)
Other	14 (8)
Educational programs for students	11 (7)
Educational programs for patients	4 (2)
Tools	4 (2)

*Note.* MGF = models, guidelines, or frameworks.

### Knowledge Contribution for the Documents

**Figure 1.**
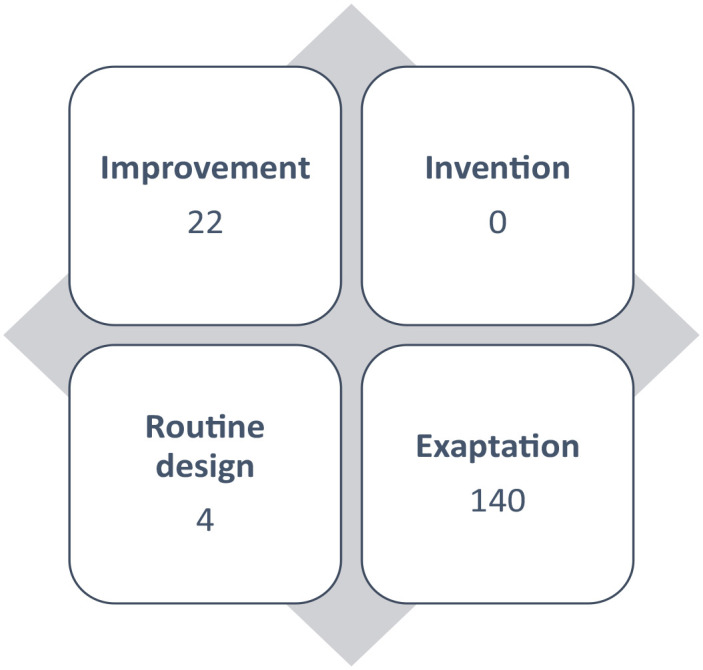
Knowledge Contribution.

The data specific to knowledge contribution were clustered by the four domains of the DSR knowledge contribution framework (Gregor & Hevner, 2013). Most contributions were clustered within the exaptation domain (*n* = 140), followed by the improvement domain (*n* = 22), then the routine design (*n* = 4), and there were no contributions classified as inventions (Figure 1).

### Nursing Models Used

**Figure 2.**
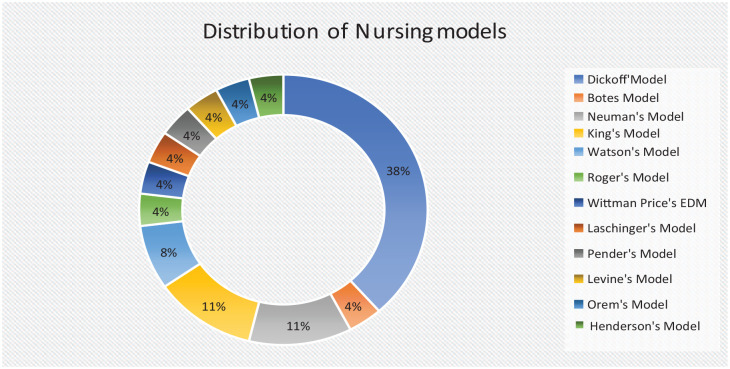
Distribution of Nursing Models.

A secondary analysis was conducted on the 26 documents that included nursing-specific grand, process, or middle-range nursing theories or models to underpin their doctoral studies. Dickoff and colleagues (1968) authored a popular process theory that was applied in most of the studies that used nursing theory. Thirteen grand nursing theories were included in the studies: Neuman’s system model (*n* = 3), King’s theory of goal attainment (*n* = 3), Watson’s theory of human caring (*n* = 2), Rogers’ science of unitary human beings (*n* = 1), Wittmann-Price’s emancipatory decision-making model (*n* = 1), Laschinger’s integrated model of nurse/patient empowerment (*n* = 1), Pender’s model (*n* = 1), Levine’s metaparadigm of nursing (*n* = 1), Orem’s self-care model (*n* = 1), and Henderson’s model of nursing (*n* = 1) (Figure 2).

## Discussion

Nursing science is a relatively young discipline, and the theoretical development of nursing knowledge is even more recent (Yancey, 2015). Nurses must contribute to the growth and development of nursing and influence the refinement of nursing science through developing, testing, and refining nursing models, theories, and metaparadigms (Barrett, 2002). As the nursing profession grows within the SSA context, nurse scientists within the region must contribute to this growth through sound research (Dobrowolska et al., 2021). This document review examined the doctoral contribution to nursing science in SSA over 5 years. The 166 documents included in this review showcase a window of the nature and focus of research by doctoral nursing students within the African continent.

Most documents in this review were predominantly from South African universities, with the majority from the UNISA. The UNISA is the largest open distance learning university in the African continent (Letseka, 2020) and a pioneer of open distance learning for doctoral programs in nursing in Africa. Due to its flexible approaches toward teaching, the UNISA justifiably has the necessary infrastructure and capacity for doctoral education in nursing within the continent. The impact of COVID-19 on the digitalization of higher education would further create opportunities for open distance learning (Zawacki-Richter, 2021), and universities such as the UNISA are crucial in enhancing the initiative. However, the notable predominance of South African universities in the documents may explain the uniformity in the doctoral programs. South African nursing doctoral degree programs are research based without any coursework (Nerad, 2011).

Coursework in doctoral education often focuses on advanced research methodology and nursing theories and models, as reflected in the doctoral nursing programs in countries such as the United States (Dobrowolska et al., 2021), creating a springboard for the introduction and application of nursing theories and models in research.

The purpose of the studies has a positive skew toward nursing practice. A research focus is often dependent on several factors, including the researcher’s personal interest, experience, expertise, and institutional capacity to engage with the study (Dobrowolska et al., 2021). The research supervisors influence the direction and focus of the studies based on their areas of specialization or expertise. A positive skew toward practice aligns with the aim of nursing science to expand and understand the human lived experience, especially concerning illness and health (Reese et al., 2016). In the current study, the focus on nursing practice may also reflect the diverse healthcare challenges within nursing practice and evidence the generic challenge in operationalizing the nursing metaparadigm within mainstream health systems in Africa.

Most of the described contributions aligned with the exaptation domain of the DSR knowledge contribution framework. The exaptation domain broadly refers to the appropriation of artifacts from other fields or disciplines to solve issues in another field—in this case, the use and application of models from other disciplines to engage with issues in nursing and healthcare. Exaptations occur when the artifacts in the field are not available or suboptimal in engaging with a lowly matured research problem (Gregor & Hevner, 2013). Non–nursing-specific theories may have been deemed appropriate for the research question and hence applied in studies. However, the low uptake of nursing models and poor integration of nursing science into mainstream healthcare, education, and research in nursing may contribute to this high number of studies applying models and frameworks from other fields (Dobrowolska et al., 2021; Hoeve et al., 2014). Outcomes from the exaptation type of contribution may not directly influence the development and refinement of nursing science, since they are already founded on theories from other disciplines. Barrett (2002) stated that nursing research that generates or tests theories from other disciplines is not nursing research. The outcome of this study chimes with the works of Parse (2015), who highlighted that most nursing studies funded by specific organizations are rarely conceptualized within nursing frameworks or theories. Rather, they are conceptualized in theories of other disciplines, with the only reference to nursing being that the nurse is a principal investigator or coinvestigator.

Interestingly, only 26 of the 166 studies applied nursing theories, models, or frameworks in their research. This figure is significantly low, especially for doctoral programs in nursing or nursing science. Intangible understanding of nursing models and theories, different views on the role of nursing science in nursing research, and the lack of knowledge and awareness of nursing theories and models have been identified as some of the reasons for the low uptake of nursing models (Bhowmik, 2022). Conversely, in line with Dobrowolska and colleagues (2021), the experience of research supervisors, institutional capacity, funding stipulations, and interdisciplinary research are drivers for adopting non–nursing-specific models and theories. However, nurse scientists have questioned the definition of doctoral degrees in nursing if they are not underpinned by nursing science, which appears to be the case from the included studies. The low uptake of nursing models in doctoral studies means that the contribution to nursing science from SSA is minimal and would explain the perpetual challenge in positioning and professionalizing nursing within the context. Parse (2015) stated that a critical mass of nurses is crucial in articulating the integration of nursing science into mainstream nursing. The already low uptake of nursing models in the included doctoral programs is indicative of the lack of interest, knowledge, or specialization related to nursing science from scholars in the region.

Nursing theories are reflecting concepts that relate to nursing principles and serve a nursing-related purpose (Havenga et al., 2014). Theories in nursing are classified as grand, middle-range, and process theories. The following grand theories were used in some of the included studies: Neuman’s system model, Rogers’ science of unitary human being, Whitman’s emancipatory decision-making model, Laschinger’s integrated model, King’s theory of goal attainment, Watson’s theory of human caring, Pender’s model of nursing and health promotion, Orem’s self-care, Roy’s adaption model, and Henderson’ model. These grand theories were initially developed and used extensively within the Western context, with some widespread application in various nursing contexts globally (Hoeck & Delmar, 2018). Inasmuch as there is evidence of locally developed models or frameworks, none of the studies applied these in their research. Such a stance is indicative of limited application and growth in locally developed frameworks.

Nurses with doctorates should change their practice and function as leaders in directing and incorporating evidence of integration into practice, education, administration, and research, including facilitating approaches toward the increased update of nursing science (Dobrowolska et al., 2021). As much as there may be few individuals in Africa with doctoral qualifications in nursing, their value may be diluted due to the limited awareness and integration of nursing science in their doctoral education. Doctoral research from SSA does not contribute substantially to nursing science, as evidenced by the low uptake of nursing models and theories and the appropriation of models from other disciplines. This finding would explain the stunting of nursing science within the African space and the challenges toward professionalizing nursing on the continent. Hoeve and colleagues (2014) argued that the professionalization of nursing is intertwined with a focus on developing nursing theory, research, and practice, which are ideally interrelated. The growth of nursing science directly influences the professionalization of nursing. The current challenges faced in the professionalization of nursing within the African context are aligned with the stunted growth and development of nursing science in the region.

We acknowledge that the included sample is not representative of the entire doctoral nursing education process or outcomes in SSA, based on the limitation of our inclusion criteria of studies in the English language and from online repositories. However, incorporated documents provide a snapshot of doctoral education in nursing in Africa. We acknowledge that this review focused on doctoral studies submitted between 2015 and 2019; at the same time, we understand that there were no significant developments in this field in Africa. Doctoral studies submitted after this time frame are most likely to reflect similar findings.

We recommend (a) an intensive review of the standards defining doctoral education for nurses in Africa, (b) a purposeful introduction to nursing science through compulsory modules on nursing theory and its integration into doctoral nursing research, and (c) focused systematic research on nursing research within SSA about nursing science. This recommendation aligns with the suggestions by Dobrowolska and colleagues (2021), who stress the urgent need to change the doctoral programs in nursing to support the advancement of nursing science. Several organizations, including the Institute of Medicine, call for a doubling in the number of doctorally prepared nurses (Broome & Fairman, 2018). However, caution should be in place regarding the program’s nature and the overall contribution to the growth of nursing science.

## Conclusion

As a relatively new discipline, nursing science faces a risk of extinction due to various factors, including the lack of clarity on the specifications of nursing science among the nurses. Doctoral research is an opportunity for nurses to demonstrate the application of grand nursing theories in the development of context-specific models, frameworks, and guidelines to further contribute to the discourse of enhanced nursing science. This document review shows a low uptake in nursing-specific theories, models, and frameworks in nurses’ doctoral education, consequently resulting in a suboptimal contribution to nursing science. Therefore, nursing science in SSA continues to be stunted with limited theoretical and practical contributions. Discourse on the professional elements of doctoral education for nurses, including the theoretical contributions, should be elevated, including exposing doctoral students to quality theoretical discourse in nursing science before and during their studies.

The following statement by Yancey (2015) comes to mind:

Failure to incorporate theories of nursing in nursing education programs will have dire consequences for the future of the discipline as nurses lose sight of what is uniquely nursing. (p. 276)
